# Incorporating epilepsy genetics into clinical practice: a 360°evaluation

**DOI:** 10.1038/s41525-018-0052-9

**Published:** 2018-05-10

**Authors:** Stephanie Oates, Shan Tang, Richard Rosch, Rosalie Lear, Elaine F. Hughes, Ruth E. Williams, Line H. G. Larsen, Qin Hao, Hans Atli Dahl, Rikke S. Møller, Deb K. Pal

**Affiliations:** 1 King’s College Hospital, London, UK; 2 Evelina London Children’s Hospital, London, UK; 3 Kings College London, London, UK; 4 Amplexa Genetics, Odense, Denmark; 5 Danish National Epilepsy Centre, Dianalund, Denmark; 6 Institute for Regional Health research, University of Southern Denmark, Odense, Denmark

## Abstract

We evaluated a new epilepsy genetic diagnostic and counseling service covering a UK population of 3.5 million. We calculated diagnostic yield, estimated clinical impact, and surveyed referring clinicians and families. We costed alternative investigational pathways for neonatal onset epilepsy. Patients with epilepsy of unknown aetiology onset < 2 years; treatment resistant epilepsy; or familial epilepsy were referred for counseling and testing. We developed NGS panels, performing clinical interpretation with a multidisciplinary team. We held an educational workshop for paediatricians and nurses. We sent questionnaires to referring paediatricians and families. We analysed investigation costs for 16 neonatal epilepsy patients. Of 96 patients, a genetic diagnosis was made in 34% of patients with seizure onset < 2 years, and 4% > 2 years, with turnaround time of 21 days. Pathogenic variants were seen in *SCN8A, SCN2A, SCN1A, KCNQ2*, *HNRNPU, GRIN2A*, *SYNGAP1, STXBP1, STX1B, CDKL5, CHRNA4, PCDH19* and *PIGT*. Clinician prediction was poor. Clinicians and families rated the service highly. In neonates, the cost of investigations could be reduced from £9362 to £2838 by performing gene panel earlier and the median diagnostic delay of 3.43 years reduced to 21 days. Panel testing for epilepsy has a high yield among children with onset < 2 years, and an appreciable clinical and financial impact. Parallel gene testing supersedes single gene testing in most early onset cases that do not show a clear genotype-phenotype correlation. Clinical interpretation of laboratory results, and in-depth discussion of implications for patients and their families, necessitate multidisciplinary input and skilled genetic counseling.

## Genetic screening: actionable information for epilepsy patients and clinicians

Screening for epilepsy-related gene variants can lead to effective, personalized treatment plans while reducing costs. UK and Danish scientists, led by Deb Pal, King’s College London, evaluated a new service within the UK that searches for genetic variants in patients that cause epilepsy. The authors assessed the impact of next-generation gene panel tests, as well as the necessary resources to make such a service effective. Genetic testing was most effective in patients with seizure onset under 2 years old (21% diagnosed) and yield even higher in neonatal-onset epilepsy (63% diagnosed). For many patients with pathogenic variants, the diagnoses allowed for recommendations on treatment or enrolment in clinical trials. The researchers found that diagnostic delay and financial burden in neonatal epilepsy could be drastically reduced with gene panel testing. The scheme was highly rated by users and patients alike.

## Introduction

Genetic testing and counseling for epilepsy is now being incorporated into everyday practice in many parts of the industrialised world.^1^ This advance has been driven by rapid discoveries in the aetiology of rare monogenic epilepsies, and technological developments in next generation resequencing (NGS).^2^ The integration of NGS testing into practice is accompanied by several challenges including clinician education, results interpretation, and counseling for patients and their families.^3^

We reflect on our experience of this transformational change from the perspective of a health service provider, specifically assessing: (a) the effectiveness and utility of NGS testing, (b) the necessary inputs and (c) areas where service improvements can be made to facilitate the transition to 'Precision' or 'Personalised Medicine'. We also asked specific questions about single vs parallel gene testing pathways based on clinician predictive ability; the relative diagnostic yield for different age of onset or epilepsy syndrome; what priorities clinicians and families identify; the resources necessary to provide an effective service, and whether NGS can save time and money^4,5^ using the neonatal epilepsy group as an example. We address these questions in the context of a review of the initial operation of a UK regional epilepsy genetics service to a population of approximately 3.5 million. To our knowledge there is limited published data from other specialist epilepsy genetics services that similarly reviews their own experience,^6^ therefore this study aims to fill a gap in that respect. However, there are several articles on the utility of genetic testing in epilepsy and published yield.^2,7–9^ Our study aims to add to the current literature and, in addition, fill in the gaps in knowledge about how to set up a tailored epilepsy genetics service, what referring clinicians and patients and families think about such a service, and the cost saving implications of performing genetic testing.

## Results

### Demographics

Ninety-six unrelated eligible patients (55 male) were referred to the service, either through the specialist outpatient clinic (*n* = 40) or directly for molecular investigation through their paediatrician or paediatric neurologist. All were consented for gene panel analysis. As this was a new service, many patients were tested years after onset or diagnosis, including one adult patient and two post-mortem. We categorized them broadly into age of onset and syndrome classes (Table 1). Sixty-four percent (49/77) were classified as drug-resistant.^10^

**Table 1:** Demographics of patients for gene panel testing

Age at seizure onset	Syndrome	Number	Age at testing median years, (range)
Neonatal (0–1 mo)	NEE	14	3.75 (0.2–16.9)
	Benign neonatal	2	0.2 (0.2)
Infantile (2–24 mo)	Infantile EE	19	7.5 (0.3–22.9)
	FS/TLE spectrum	4	6.1 (1.3–18.3)
	Infantile spasms	11	6.5 (0.5–12.2)
Childhood (>2 y)	NFLE/SHE	6	13.7 (5.6–17.6)
	Generalised (LGS-like)	9	15.1 (3.4–19.9)
	Early-onset absence	4	7.45 (1.4–14.7)
	Epilepsy-Aphasia spectrum	11	10.8 (7.3–17.2)
	Familial focal epilepsy	8	10.45 (4.0–14.5)
	Refractory focal epilepsy	8	9 (4.4–17.4)
Total		96	**7.5 (0.2–22.9)**

*NEE* neonatal epileptic encephalopathy (epilepsy with onset between birth and 3 months of age),

*IEE* infantile epileptic encephalopathy (epilepsy with onset between 4 and 12 months),

*GEFS* generalised epilepsy with febrile seizures (https://www.epilepsydiagnosis.org/syndrome/fbp-overview.html),

*TLE* temporal lobe epilepsy (https://www.epilepsydiagnosis.org/syndrome/other-familial-temporal-lobe-overview.html),

*NFLE* nocturnal frontal lobe epilepsy also known as *SHE* sleep-related hypermotor Epilepsy,^37^

*LGS* Lennox–Gastaut syndrome (https://www.epilepsydiagnosis.org/syndrome/lgs-overview.html)

### Identified variants

Seventy-four of our 96 patients had previous array comparative genomic hybridisation (aCGH) performed (77%), of which 16 (22%) had an identified benign chromosomal rearrangement. The remainder had no detected rearrangement and a normal chromosomal complement. Patients with pathological findings on the aCGH do not tend to make their way into our clinic. In fact, only three patients out of forty-four referred for aCGH by one of the three local clinicians we work with were found to have a pathogenic chromosomal rearrangement by the local laboratory (ViaPath) and were not referred on to our service: one showing Angelman’s syndrome; one Klinefelter’s syndrome; and one showing 9q34 deletion. However, the match between the epilepsy phenotype and the chromosomal rearrangement is not conclusive in any of these three cases so none of these can be considered completely 'solved'.

For NGS panel testing, 11 patients were tested on the original Childhood Epilepsy panel containing 45 genes (CHE-45); 11 on the CHE-76 (Childhood Epilepsy panel containing 76 genes); 49 on CHE-85 (Childhood Epilepsy panel containing 85 genes), and 23 on CHE-102 (Childhood Epilepsy panel containing 102 genes); 2 patients were referred to the epilepsy genetics service with existing positive gene panel results from another provider. The gene panel itself was designed by the following co-authors: RSM, DKP and HAD. The criteria for including a gene on the panel were that it should have been reported more than once in patients with monogenic epilepsies. The selection of genes on the panel was regularly evaluated and updated. The panel included of targeted capture of all exons and at least five base pairs of flanking intronic sequence of the selected genes.

The overall target coverage of the genes on the Amplexa CHE-46 panel was 95–97%; hence, 3–5% of the regions were not analyzed, and some variations may have been missed, while the average target coverage for the larger three panels was 98–99.5%. The regions missed were more or less identical across the different samples, i.e., regions difficult to amplify due to high GC content, repeat elements, or regions with homology in other parts of the genome.

Amplexa Genetics reporting follows the ACMG guidelines. However, there is an argument that these guidelines are not very suitable for conditions with variable penetrance (which many types of epilepsy have been shown to have). As our knowledge and understanding of epilepsy genetics is still limited, they do also report Class II (benign) variants, and this allows us to monitor them in case our understanding changes in the future. When we receive a report from Amplexa, we then compare that with our understanding of the phenotype to check whether this fits with the clinical picture. This often leads us to re-grade classifications of variants reported. If, however, we are still uncertain, we will request assistance from experienced colleagues in the field. Parental segregation may also lead to re-classification of variant class if the results fit with the phenotype or family history, e.g., 95% of SCN1A variants causing SMEI will be de novo. Parental segregation was deemed necessary when a class IV variant (defined as per the ACMG 2015 Guidelines^11^) or above was identified in the child, or a Class III variant was identified and it was in a gene that seemed to match with the child’s phenotype and/or family history, or in genes where de novo variants are usually pathogenic.

61% of patients (*n* = 59) had one or more variants (single nucleotide variants only) reported: 31 had only benign variants; 9 had variants of unknown significance (VUS), and 19 had variants judged to be of pathogenic significance. The average number of any variant, not just pathogenic, increased in line with the expansion in size of the gene panel (CHE-46: 1.3; CHE-102: 1.8) indicating the additional burden of clinical interpretation.^12^ We were constrained in our ability to retest panel negative cases because the testing was done under clinical auspices and therefore no patient with initial negative results were retested on a larger panel in this study. This means that the stated diagnostic yields probably underestimate what could have been achieved if everyone had been tested on the most up to date panel. The average turnaround time for results was 21 working days, less when no variants were seen (18 days) because Sanger validation was not necessary, and slightly more when parental segregation and new sample collection were necessary.

The pipeline used by Amplexa Genetics to establish pathogenicity of variants does indeed resemble other genetic testing NGS models.^11–13^ The panels used were designed to cover all coding exons and exon-intron boundaries of the included genes, including an additional 10 bp of the introns. Sequences were aligned to hg19 using the Torrent Suite (ThermoFisher) and SNPs with a read depth ≥ 20 and variant allele frequency of ≥0.25 were called using the Strand NGS software. Rare or low frequency variants were evaluated in an internally developed pipeline. Included in this evaluation were literature and database searches like Human Gene Mutation Database (HGMD), Exome Aggregation Consortium (ExAC) database, the Genome Aggregation Database (gnomAD). Synonymous variants and variants in autosomal dominant genes which had been observed more than three times or in homo-/hemizygous state in the ExAC/gnomAD database were excluded in severe epileptic encephalopathy (EE) cases. All variants were submitted to prediction tools—predictions on protein level were obtained from dbNSFP Functional Predictions and Cores 3.0 database while the variants were submitted to bioinfomatic software tools, e.g., NNSplice and ESEfinder for predictions on transcriptional level. The ACMG guidelines were applied to the resulting variants.^11^ Pathogenic variants are listed in Table 2: *SCN8A* (*n* = 4) and *SCN2A* (*n* = 3) were the two most commonly implicated genes. Two pathogenic variants were observed in *SCN1A* but not in typical *SCN1A*-associated generalised epilepsy with febrile seizures or Dravet syndrome cases. Variants of unknown significance were detected in *GABRA5, SCN8A, CHRNB2, RYR3, HNRNPU, CACNA1A, SPTAN1, PIGA, KCNQ3, SLC2A1, NPRL3 and CHRNA4* (Table 3).

**Table 2:** Pathogenic, or likely pathogenic variants in 18 cases

Phenotype	Gene	Variant(s) c.DNA change	Amino Acid change	Inheritance	SIFT prediction	POLYphen prediction	gnomAD prevalence	Variant published
NEE	*SCN8A*	c.3979A>G	p.Ile1327Val	Unknown	DAM	DAM	0	17,18
NEE		c.4883T>G	p.Leu1628Trp	Unknown	DAM	DAM	0	No
IEE		c.5630A>G	p.Asn1877Ser	*De novo*	DAM	DAM	0	19–22
FFE		c.5615G>A	p.Arg1872Gln	Paternal (mosaic)	DAM	DAM	1/246048	23
BFNIS	*SCN2A*	c.623T>A	p.Val208Glu	Paternal (aff)	DAM	DAM	0	8
NEE/MMPSI		c.640T>C	p.Ser214Pro	*De novo*	DAM	DAM	0	14 ^c^ single case, ClinVar, 372557
NEE		c.1312G>A	p.Glu438Lys	Unknown	DAM	DAM	0	single case, ClinVar 207057
FFE	*SCN1A*	c.4871T>A	p.Leu1624Gln	Maternal (aff)	DAM	DAM	0	No
EO-ABS^a^		c.4943G>A	p.Arg1648His	*De novo*	DAM	DAM	0	No
BFNIS	*KCNQ2*	c.476G>A	p.Gly159Glu	Paternal	DAM	DAM	0	24
NEE		c.1678C>T	p.Arg560Trp	*De novo*	DAM	DAM	0	25
NEE, LD	*HNRNPU*	c.1681delC	p.Gln561SerfsTer45	*De novo*	DAM	DAM	0	26 ^c^
	*CHRNA4* ^*b*^	c.1454G>A	p.Arg485Gln	Maternal	DAM	DAM	40/177598	3 cases ClinVar 197690 (2 VUS)
ABPE-ESES	*GRIN2A*	c.2179G>A	p.Ala727Thr	Paternal	DAM	DAM	0	^27^
IEE	*SYNGAP1*	c.1766T>A	p.Ile589Asn	*De novo*	DAM	DAM	0	No
NEE	*STXBP1*	c.1282C>T	p.Gln428Ter	*De novo*	DAM	DAM	0	No
Gen sz, DD, ASD	*STX1B*	c.563dupA	p.Gln189AlafsTer5	Unknown	DAM	DAM	0	No
IS, DD, VI	*CDKL5*	c.2177_2168delCTTTCCATGAinsAATGTGTCAAC	p.Ser726Ter	Unknown	DAM	DAM	0	No
FS clusters^a^	*PCDH19*	c.344_345insT (exon 1)	p.Val117GlyfsTer109	*De novo;* mosaic male	DAM	DAM	0	No
NEE	*PIGT*	c.709G>C (homozygous)	p.Glu237Gln	Recessive	BEN	DAM	16/243502 (N/A)	No

*NEE* neonatal epileptic encephalopathy, *IEE* infantile epileptic encephalopathy, *BFNIS* benign familial neonatal-infantile seizures, *MMPSI* malignant migrating partial seizures of infancy, *EO-ABS* early onset absence seizures, *FFE* familial focal epilepsy, *heat-sens* heat sensitive seizures, *LD* learning disability, *ABPE-ESES* atypical benign partial epilepsy with electrical status in slow-wave sleep, *DD* developmental delay, *ASD* autism spectrum disorder, *VI* cortical visual impairment, *DAM* damaging, *BEN* benign, *TOL* tolerated

^a^see text for details

^b^CHRNA4 likely to be a modifier in this patient

^c^Same patient, published previously

**Table 3:** Variants of unknown significance

Phenotype	Gene	Variant(s) c.DNA change	Acid change	Inheritance	SIFT prediction	POLYphen prediction	gnomAD prevalence	Comments
FFE	*GABRA5*	c.86 + 1G>A	–	Paternal	–	–	3/277182	Broken splice site
LGS	*SCN8A*	c.659G>A	p.Arg220His	Unknown	DAM	DAM	0	predicted
NEE, LD, ASD^a^	*CHRNB2*	c.1378C>G	p.Arg460Gly	Maternal	TOL	DAM	108/277170	3 cases ClinVar 191352 (2 VUS; 1 Likely benign)
ABS, regression	*RYR3*	c.573A>G	p.Ile191Met	Maternal	BEN	DAM	0	No
IEE	*HNRNPU*	c.2197_2199delAGG	p.Arg733del	Unknown	DAM	DAM	0	No
NEE	*CACNA1A*	c.1854G>T	p.Leu618Phe	Unknown	DAM	DAM	0	No
NEE	*SPTAN1*	c.6178G>A	p.Glu2060Lys	Unknown	BEN	BEN	0	
	*PIGA*	c.1A>G	p.Met1?	Unknown			0	
IEE	*RYR3*	c.4471G>A	p.Asp1419Asn	Unknown – not maternal	BEN	DAM	0	Disease causing in Mutation Taster http://www.mutationtaster.org/c gi- bin/MutationTaster/MutationTas ter69.cgi?new_base=A&transcrip t_stable_id_text=ENST00000389 232&position_be=4471&gene=R YR3&transcript_stable_id_radio=ENST00000389232&sequence_ty pe=CDS
>2 FOC	*SLC2A1*	c.586C>G	p.Pro196Ala	Maternal	BEN	DAM	0	
FOC	*NPRL3*	c.103C>G	p.Pro35Ala	Unknown	BEN	BEN	1/30884	Disease causing in Mutation Taster^49^
	*CHRNA4*	c.77-8C>T		Unknown	BEN	BEN	0	

*FFE* familial focal epilepsy, *LGS* Lennox–Gastaut syndrome, *FOC* focal epilepsy, *DAM* damaging, *BEN* benign

^a^This variant was considered a potential modifier, due to the severe presentation

### Variant yield

The yield varied according to age of seizure onset—Table 4 shows results by patient and, if a patient has several different variants they are classified by their most 'serious' ranked variant (pathogenic > VUS > benign). The 59 patients with at least one variant (benign, VUS and pathogenic included) had a total of 54 benign variants amongst them (17 patients had more than one benign variant and 6 had one or more benign variants plus a VUS or pathogenic variant as well); 9 variants of unknown significance; and 20 pathogenic variants (one patient had two variants in two different genes). 12 of the variants were Class 4 and 7 were class 5, as per the ACMG guidelines.^11^ The diagnostic yield, defined as the percentage of cases “solved” by NGS panel testing was highest in the neonatal onset epilepsies (63%), intermediate in the remaining first 2 years of life (21%), and lowest when onset was later (4%). The diagnostic yield was 23% among drug resistant cases. Clinicians attempted gene prediction (by informed guesses) in 33 cases, and were correct in five (15%): *SCN1A, PCDH19*, *GRIN2A, CDKL5, SCN2A*.^9^

**Table 4:** Variant yield by age of onset and epilepsy syndrome

	Patients with					
Age at seizure onset	no variants	only benign variants	VUS	pathogenic variants	Total	Diagnostic yield
0–1 m	1	3	2	10	**16**	63%
2–24 m	14	10	3	7	**34**	21%
IEE	5	7	3	4	**19**	21%
FS/TLE	2	0	0	2	**4**	50%
IS	7	3	0	1	**11**	^a^9%
>2 y	22	18	4	2	**46**	4%
NFLE/SHE	4	2	0	0	**6**	0%
GGE	4	3	2	0	**9**	0%
EOABS	0	4	0	0	**4**	0%
ESES	5	5	0	1	**11**	9%
FFE	4	2	1	1	**8**	13%
DRE-FOC	5	2	1	0	**8**	0%
Grand total	37	31	9	19	**96**	20%

^a^one further case was subsequently solved through whole genome research investigation

### Impact

In 63% of cases with pathogenic variants, the results had an immediate implication for treatment. Most involved ion channel subunit genes such as *SCN1A, SCN2A, SCN8A, KCNQ2*, leading to recommendations about Na + blocking antiepileptic drugs in 10 cases. Two cases with acetyl-choline receptor subunit variants that were suspected phenotype modifiers (*CHRNA4, CHRNB2*) were offered experimental nicotine therapy.^14^ It should be noted that the patient with the *CHRNB2* VUS did not have his treatment altered because of this VUS. However, as we suspected it to be a phenotype modifier, he was offered the chance to try experimental nicotine therapy as an adjunctive treatment, to see if that had any impact on his seizures. One-quarter of cases were entered into a registry or research study. The families with pathogenic variants were offered expert genetic counseling: in six cases (31%) an additional affected relative was diagnosed.

### Workshop and surveys

19 paediatricians and epilepsy nurses attended the workshop and all offered feedback. 100% agreed that the workshop was excellent and they were likely to change their practice going forward. We received 10 survey responses from families (25% response), and six from clinicians (40%). Both the outpatient and molecular diagnostic components of the service were rated as good or excellent (100%) by clinicians. Families also rated our services highly and 100% would recommend to friends and family (Table 5).

**Table 5:** Referring clinicians’ opinions and families’ views of the epilepsy genetics service

Clinicians’ opinions	
Do you think that genetic testing..	Yes
..helped you to confirm or refine an existing or suspected clinical diagnosis?	83%
..has allowed a diagnosis to be made earlier than with clinical and EEG data alone?	83%
..saved your patient from additional investigations?	50%
..results altered your treatment and/or management approach?	67%
..results prevented the prescription of drugs that could have worsened the epilepsy?	17%
..was helpful in providing an explanation of the underlying disease for the family?	83%
Families views	
Question	Strongly/Agree
How helpful was genetic testing in giving you a cause for your child’s Epilepsy?	70%
Did the healthcare professionals give you enough opportunity to ask questions?	100%
Did the healthcare professionals explain things in a way you could understand?	100%
How helpful did you find it to attend the specialist outpatient clinic?	100%
How likely are you to recommend our service to friends or family who need similar care?	100%

### Investigational cost

We retrieved complete records for 16 neonatal epilepsy patients. Total investigation costs ranged from £5094 to £15,622, average £9362, with more than 75% of the costs allocated to neuroimaging and videoEEG-telemetry. In multiple linear regression, we found statistically significant and independent correlation only between diagnostic delay and cost of previous genetic tests (*p* = 0.011).

Prior single gene testing among this sample included Fragile-X (*FMR1*), Ataxia-Telengectasia (*ATM*), Niemann-Pick C (*NPC1*, *NPC2*), spinal muscular atrophy (*SMN1, SMN2*), Prader-Willi syndrome (15q11.2-q13), myotonic dystrophy (*DMPK*), ARX, atypical Rett syndrome (*CDKL5*), and Glutaric aciduria Type 1 (*GAT1*). Because both MRI and EEG can be performed for disease monitoring as well as diagnosis, we excluded these and focused on the remaining laboratory analyses performed on blood, urine and cerebrospinal fluid (CSF) samples. We found that two-thirds of these costs (total average per patient: £2004) were made up of array CGH and single gene tests, as well as metabolic investigations and invasive lumbar puncture. The delay between epilepsy onset and diagnosis ranged from 83 days to 17 years (median 3.4 years). Consequently, we calculated that if all neonatal epilepsy patients underwent NGS panel testing as part of their first line investigations, their theoretical total investigational costs would have averaged £2838, which is £6524 less (70%) than the actual average cost.

## Discussion

NGS panel testing in epilepsy is largely effective and useful, and has particular strengths for early onset epilepsies. The high diagnostic yield in the neonatal (63%) and infant (21%) onset groups is unprecedented. We do not think there is any one answer as to why the yield was so high, however only selecting the most appropriate patients for testing and having a good panel design are of course very important factors.

There is a significant impact on treatment and risk counselling for the majority of genetically diagnosed cases.^2^ Families put a high value on exploring the implications of the results for their child and family; and referring clinicians appreciated the quality of clinical interpretation and rapid turnaround time.

The inputs required are substantial and complex: in our context, they were based on an existing integrated tertiary and secondary level regional epilepsy service, and relied on an educated referral base to select appropriate cases, an expert multidisciplinary team for interpreting variants with clinical features, and the skills of a specialized genetic counselor to translate findings into tangible benefits for families.

There is also a potential for huge reduction in investigation burden, cost and delay, taking into account the priorities of users and referrers.

### Utility and effectiveness

#### Diagnostic yield and clinical impact

Yields of 10–48.5% have been reported from diagnostic NGS panels consisting of 36–265 target epilepsy genes,^7,9,15–20^ with a higher diagnostic yield in children under 2 years at seizure onset. We found patients with pathogenic variants in the most common epilepsy genes *SCN8A* (*n* = 4), *SCN2A* (*n* = 3), *SCN1A* (*n* = 2), *KCNQ2* (*n* = 2) and *STXBP1, GRIN2A, CHRNA4* (*n* = 1 each), accounting for 70% of all presumed disease-causing variants (Table 2). In all living cases involving Na or K channel mutations, recommendations or changes were made to antiepileptic medications. 9% of cases were entered into a clinical trial; 26% of cases were entered into a phenotype registry or study awaiting future trials, and families were introduced to online patient groups. Additionally, one quarter of patients had another relative diagnosed following their diagnosis. The rapid turnaround time of 21 working days (14 days for urgent cases) means interventions could be started in sufficient time to theoretically modify disease course or prevent complications, although the evidence base for such therapies is yet to be established.^21^ In addition, we found presumed pathogenic variants in epilepsy genes that have not been well characterized including *HNRNPU*, and the recessive *PIGT* (homozygous). 8 of the 20 pathogenic variants have previously been published^7,8,15,22–32^ a further 3 are listed in ClinVar (Table 2); while 9 are novel.

#### Single vs parallel gene testing

The philosophy of parallel testing or 'gene-first', in patients where a genetic cause is suspected but there is extensive genetic heterogeneity, is vindicated by clinicians’ limited ability to predict results, and by some remarkable surprises. Clinician prediction was not often attempted and we suspect this is because of the extreme genetic heterogeneity, pleiotropy, reduced penetrance and variable expression in infantile onset epilepsies, these factors providing the rationale for parallel gene testing.^33,34^ The cases in which prediction was attempted reflect examples where there is better known genotype-phenotype correlation. There are for example, some more specific clinical features that are characteristic of one, or a handful, of genes: clustering of febrile seizures (*PCDH19*); temperature sensitivity (*SCN1A*); etc. We discuss two case examples involving patients with pathogenic mutation in these genes in the discussion section.

Still there were many surprises as evidenced by the poor prediction rate. The full phenotypic spectra of many epilepsy genes are currently being reported in the literature; as part of our continuing clinician education for referring clinicians, we aim to disseminate this new knowledge to ensure that patients are accurately selected for genetic testing. The following three cases deserve discussion because they demonstrate the strong clinical foundation necessary for genetic testing in epilepsy.

The first was a 7-year-old child with early-onset (3 years) drug-resistant absence seizures preceded by multiple febrile seizures; her mother noted the absences were sensitive to high temperature. Her father had drug-responsive juvenile onset absence epilepsy. aCGH showed a paternally inherited 15q13.3 deletion, which explained the familial susceptibility to absence seizures, but not the daughter’s early age of onset, drug resistance, febrile seizures or heat sensitivity. Gene panel testing then revealed a de novo mutation in *SCN1A*, p.Arg1648His (Table 2).

The second had an onset of Lennox-Gastaut like symptoms in the first year of life, with severe learning difficulties including developmental regression of language and motor function at the age of 3. He had a pattern of nocturnal motor seizures clustering over several days, repeating three times per month, and was drug-resistant. NGS panel results showed pathogenic variants in *HNRNPU* (de novo) and *CHRNA4* (inherited); the former explaining his overall phenotype, the second explaining his clustering nocturnal motor seizures. A trial of transdermal nicotine significantly reduced his nocturnal motor seizures and improved his daytime communication and functioning.^14^ Both this case and the *SCN1A* case exemplify how 'second hits' can modify a seizure phenotype and also act as a focus for therapeutic modulation.

The third case had severe clusters of infantile convulsions continuing for 48–72 h and recurring every few months with intercurrent febrile illness; at age 11 years he became seizure free on levetiracetam and now attends college. His clinical features resembled the seizure phenotype described in Epilepsy with Mental Retardation Limited to Females.^35^ NGS panel testing surprisingly revealed a mosaic heterozygous mutation in *PCDH19*. There are very few reported cases in males, and the genetic mechanism remains obscure.^36^

In the older age group (seizure onset > 2 years), the diagnostic yield was relatively low (4%). One reason is that far fewer genes have been discovered in later onset epilepsies, and this should prompt us towards more concerted efforts in collaborative gene discovery, especially in the focal epilepsies. However, it is likely that many of these later-onset epilepsies have a more complex aetiology and so even when we discover some of the associated genes, their impact on disease development will probably be modest and show wide variability of penetrance and expression amongst affected individuals.

Genes for autosomal dominant sleep-related hypermotor epilepsy, although among the first discovered (*CHRNA4, CHRNB2, CHRNA2, KCNT1, DEPDC5, CRH, PRIMA1*) still only explain approximately 10% of cases.^37^ Unfortunately, none of our five tested patients carried a causative mutation, suggesting that genetic testing is not cost-effective in differentiating nocturnal motor phenomena in adolescents. Only recently, new genes for familial focal epilepsy (FFE) have been reported from the *GATOR1* pathway (*DEPDC5, NPRL2, NPRL3*) and these were missing from earlier versions of the gene panel CHE-46, CHE-76, CHE-85). While we speculate that some of our FFE patients might have tested positive, we note the low (0.8–12%) current yield in sporadic and FFE cases.^38^

We also noted the low yield for children with infantile or epileptic spasms. Infantile spasms are aetiologically heterogeneous: tuberous sclerosis is the most common single cause, followed by hypoxic-ischaemic injury, stroke and brain malformations, and 70% of cases have abnormal MR imaging.^39^ In a recent study of 44 unsolved infantile seizures cases, 7% had a de novo chromosomal rearrangement, and pathogenic mutations were revealed by trio exome sequencing in 28% of the remainder, suggesting that the diagnostic yield can be significant in fully investigated unsolved cases.^40^ Among our nine unsolved cases, a complete imaging, cytogenetic and metabolic screen had only been completed in one, suggesting room for better workup of these cases prior to NGS panel testing.

### Necessary inputs

#### Clinical interpretation

Variant interpretation is not always straightforward, and requires close cooperation between molecular geneticist, bioinformatician, neurologist and genetic counsellor. We dealt with a large volume of benign (*n* = 54) or VUS (*n* = 11), which represents a substantial burden for clinical interpretation as well as a source of uncertainty for families. VUS arise for a number of reasons, e.g., inadequate bioinformatic prediction, lack of functional data, missing segregation, or incomplete knowledge of genotype-phenotype correlation. In this scenario, segregation information on a novel variant only contributes to diagnostic certainty when there is confidence about the bioinformatic prediction and the associated epilepsy phenotype. If the evidence is scant, then proving that the change is de novo, or segregates with disease in an affected parent will, in reality, make very little difference to the patient or family until further evidence establishes the VUS as likely pathogenic, or benign. Without expert interpretation, clinicians may be vulnerable to pitfalls such as over-interpreting variants as mutations or vice-versa,^41^ and wrongly assigning pathogenicity to heterozygous variants in recessive conditions.

#### Clinician education and health structure

Clinicians who understand the benefits and limitations of the service are able to offer it most effectively to the right patients. Our educational workshop was very useful in this regard, and most referrals that we received from workshop participants were appropriate and properly worked up beforehand. Without this hierarchical structure, there is the possibility of bypassing guidelines on investigation and wasting resources. However, clinical education is an ongoing process and continuing feedback on outcomes and beneficial impacts are probably necessary to sustain and grow referrals and appropriate NGS requests.

#### Genetic counseling

Despite universal access to the internet, many families have limited understanding of the principles of human genetics and require clear and relevant information, relayed in the context of their own situation before they can make an informed decision about genetic testing. Genetic counseling is the process of helping people understand and adapt to the medical, psychological, and familial implications of genetic contributions to disease.^42^ The genetic counselor is therefore ideally placed to discuss with the family: facilitating adaptation to their child’s condition, discussing the process and implications of genetic testing, as well as promoting informed choices, for now and in the future (e.g., family planning). A large proportion of genetic epilepsies are as a result of de novo mutations, and so cascade testing for the wider family is often not necessary. However, as germline mosaicism is now thought to be more common than it was originally,^43^ the possibility of prenatal testing in any future pregnancies is always discussed.

### Re-engineering services for precision medicine

#### Clinician and family feedback

Clinicians valued the new specialist service, perceiving it helpful for diagnosis, management and counseling, and 50% believed it had saved additional investigations. Referrals increased over the course of the study, indicating an unmet need in the population. Families also found the experience of genetic counseling and testing helpful, regardless of whether their child’s case was solved or not. This feedback points to the need for informed and unhurried discussion around genetic testing, something that cannot be currently achieved in the current constraints of a general neurology clinic.

#### Cost saving

Clinician perceptions of cost-saving are supported by the analysis of neonatal epilepsy data, showing that investigation costs could be reduced by two-thirds by ordering an NGS panel earlier in the pathway, which has been noted before.^2,4,5^ This might also reduce the median diagnostic delay from 3.43 years to 21 days and feasibly allow the early use of disease-modifying drugs. However, true cost-savings are likely to be less than the theoretical and would need to be calculated using a prospective study design, preferably with a non-NGS tested concurrent control group. Such calculations may need to be repeated as technology evolves. Nevertheless, guideline revision requires consensus and commitment from multiple organizational stakeholders.

### Limitations

While a prospective design has many advantages in terms of selection bias, there are a couple of limitations of this study. First, because our clinical pathway separates children with primary epilepsy from all children with early-onset seizures, and requires a routine workup to exclude lesional and some metabolic causes as well as excluding single gene testing for *SCN1A* and *SLC2A1*, the results may not be generalizable to other health care contexts. Second, our diagnostic yield concealed some variability because of the evolution of the gene panel over the period of study, reflecting the fast pace of gene discovery—this might have led to some under-diagnosis of patients using earlier panels.

## Conclusion

NGS-based genetic testing has high clinical utility in children with epilepsy onset before 2 years or in drug-resistant or familial cases. The impacts are numerous and range from treatment change to risk counseling, and potential recruitment to clinical trials as new experimental therapies become available. A successful service requires strong engagement from secondary health care providers, an existing framework for specialist referral and investigation, substantial collaboration between clinicians and scientists for variant interpretation, as well as expertise in genetic counseling and flexibility in communicating with and meeting the evolving needs of families. To make the best of any innovation in medicine, health care organizations need to be open to change and reconfiguration of resources to benefit patients and their families.

## Methods

### Ethics

(a) Methods were performed in accordance with relevant regulations and guidelines and (b) methods were approved by The Great Ormond Street Hospital/Institute of Child Health Research Ethics Committee (reference number: 09/H0713/76).

### Population

We collected prospective data related to genetic testing on 96 patients referred to the King’s Health Partners epilepsy genetics service for molecular diagnostic testing, between November 2014 and September 2016. The service is provided to the southeast region of England, a population of approximately 3.5 million including the south-east of London. The region includes two teaching hospitals with tertiary paediatric neurology departments (King’s College Hospital NHS Trust and Evelina London Children’s Hospital) and eleven district general hospitals in which there is a general paediatrician with a special interest in epilepsy. Medical services are state-run and organized through a regional clinical network with common management guidelines for epilepsy.^44^ Patients are seen first at their district general hospital before being referred, if appropriate, for a tertiary specialist opinion either at one of the two tertiary centres or in a regional specialist epilepsy clinic.

The epilepsy genetics service comprises two components: a specialist clinic run by a paediatric epileptologist with a research interest in genetics (DKP), a genetic counselor (SO) and clinical fellow (ST, RR); and a molecular genetic diagnostic service using an NGS epilepsy panel (LHGL, QH, HAD), with clinical interpretation by the whole team.

### Pathway

In the absence of consensus guidelines, we considered patients suitable for genetic testing with either early-onset (<2 years) epilepsy, treatment resistant epilepsy of unknown cause, or familial epilepsy where the genetic cause was unknown. Two of our patients sadly died during the testing process. As a rule, we only considered patients with epilepsy as their primary diagnosis, rather than patients with intellectual disability (ID) or autism (ASD) who had seizures as part of their phenotype. This is because our service is part of the epilepsy service, whereas patients with primary ID or ASD who also have seizures do not usually use our pathway, unless they have a relevant family history. Patients followed one of three pathways for genetic testing: either being seen (i) in the specialist epilepsy genetic clinic, as above (*n* = 40); (ii) by a paediatric neurologist (*n* = 7) or paediatric epileptologist (*n* = 37) at one of the two tertiary centres; or (iii) seen by a general paediatrician (*n* = 12) with a special interest in epilepsy at a district general hospital, with referrals made in discussion with their linked paediatric epileptologist. Patients were recommended to have completed routine aetiological investigations as per regional guidelines (EEG, MRI, metabolic as necessary), and the clinician was asked to complete a proforma summarizing the electroclinical phenotype, epilepsy syndrome, age at seizure onset, drug response, results of previous investigations, and clinical prediction of candidate gene. We collected aCGH data in cases where it had been performed. Children with suspected typical Dravet Syndrome (OMIM 607208) or Glut-1 Deficiency syndromes (OMIM 606777) undergo single gene testing and were not included here; patients with brain malformations are tested on a separate gene panel and also not discussed here.

At the outpatient visit, we spent approximately 1 h with each new patient. The paediatric epileptologist and genetic counsellor took a detailed clinical and genetic history and performed a neurological examination on the affected child. Patients were operationally categorized into broad epilepsy syndromes (Table 1) because many did not fit into the International League Against Epilepsy classification of epilepsy syndromes.^45^ The genetic counselor then discussed the possibility of NGS panel testing, and if the family were interested, proceeded to explain: the process; benefits and limitations; potential outcomes and what they might mean; discussed any issues of concern that might arise around results, obtained written informed consent (using the appended consent form) prior to the start of this study, and planned for follow-up.

### Education

We held a half-day educational workshop aimed at regional paediatricians and epilepsy nurses, to discuss which patients were suitable for testing, which test to choose and how to obtain informed consent. We designed the educational workshops along evidence-based lines for effective learning, using case-based simulations in small groups.^46–48^ After the workshop, attendees gave anonymous feedback indicating that 100% of them were 'likely' or 'very likely' to change their practice. We circulated proposed guidelines for genetic testing to the group which were agreed in consensus. Following this, in actual practice we have seen the number of referrals increase and that most referrals meet our published guidelines. Furthermore, the number of new referrers has increased and as we provide email feedback to every referrer, appropriateness is also improving amongst new referrers. Additionally, we posted separate information for clinicians and families on our website www.childhood-epilepsy.org.

### Gene panel

We used the Amplexa Genetics epilepsy gene panel CHE-46 (46 epilepsy genes) at the start of the service,^7^ which was updated to CHE-76, CHE-85 and CHE-102 during the study period in light of new gene discoveries (by DKP, RM, HAD), (Table 6). To identify putative disease-causing variants, we performed targeted NGS of 46–102 epilepsy genes in four successive panels (January 2014—January 2016). The criteria for including a gene on the panel were that it should have been reported more than once in patients with monogenic epilepsies. The genes included on the CHE-46 panel were: *ALDH7A1, ALG13, ARHGEF9, CACNA1A, CDKL5, CHD2, CPA6, DEPDC5, DNM1, GABRA1, GABBR1, GABBR2, GABRB3, GABRD, GABRG2, GNAO1, GRIN1, GRIN2A, GRIN2B, HCN1, HDAC4, HNRNPU, IQSEQ2, KCNA2, KCNQ2, KCNQ3, KCNT1, KCTD7, LGI1, MBD5, PCDH19, PLCB1, PNPO, PRRT2, SCN1A, SCN1B, SCN2A, SCN8A, SLC25A22, SLC2A1, SLC35A3, SPTAN1, STX1B, STXBP1, SYNGAP1*, and *TBC1D24*; additionally for CHE-76: *ADSL, ATP1A2, ATP1A3, ATRX, CHRNA2, CHRNA4, CHRNB2, GABRA5, GAMT, GATM, MECP2, MEF2C, MTOR, PIGA, PIK3AP1, PNKP, POLG, PURA, RYR3, SLC25A2, SLC6A1, SLC6A8, SLC9A6, SMARCA2, TCF4, UBE3A*; and additionally for CHE-85: *CLCN2, CNKRS2, FASN, FOXG1, HDAC4, HNRNPU, HUWE1, KCNH5, KCTD7, MBD5, PIGO, PIGT, RELN, SIK1, SLC13A5, SLC35A2, SLC35A3, SLC6A8, SLC9A6, ZDHHC9*; and for CHE-102: *CACNB4, CUX2, EEF1A2, GRIN2D, KANK1, KCNB1, KCNMA1, KIAA2022, NPRL2, NPRL3, PIK3R2, ST3GAL3, SZT2, WWOX*. aCGH, where performed, was conducted using an oligonucleotide array with ~60,000 probes across the genome. Paternity testing was not performed.

**Table 6:** Gene panels used in this study, categorized by gene function

Ion transport	Neuro-transmitter related	Gene expression	Scaffolding and trafficking	Intracellular signalling	Other functions
*ATP1A2*	*CHRNA2*	*ARX*	*CNKSR2*	*DEPDC5*	*ADSL*
*ATP1A3*	*CHRNA4*	*ATRX*	*DNM1*	*GNAO1*	*ALDH7A1*
*CACNA1A*	*CHRNB2*	*CHD2*	*IQSEC2*	*MTOR*	*ALG13*
*CACNA1H*	*GABBR1*	*CUX2*	*KANK1*	*NPRL2*	*ARHGEF9*
*CACNB4*	*GABBR2*	*EEF1A2*	*KIAA2022*	*NPRL3*	*CDKL5*
*CLCN2* ^a^	*GABRA1*	*FOXG1*	*PCDH19*	*PIK3R2*	*CPA6*
*HCN1*	*GABRA5*	*HDAC4*	*PIGA*	*PLCB1*	*FASN*
*KCNA2*	*GABRB3*	*HNRNPU*	*PIGO*	*RYR3* ^a^	*GAMT*
*KCNB1*	*GABRD*	*HUWE1*	*PIGT*	*SIK1*	*GATM*
*KCND2*	*GABRG2*	*MBD5* ^a^	*RELN*		*PIK3AP1*
*KCNH5*	*GRIN1*	*MECP2*	*SPTAN1*		*PNKP*
*KCNH8*	*GRIN2A*	*MEF2C*	*STX1B*		*PNPO*
*KCNMA1*	*GRIN2B*	*PURA*	*STXBP1*		*POLG*
*KCNQ2*	*GRIN2D*	*SMARCA2*	*TBC1D24*		*ST3GAL3*
*KCNQ3*	*NRXN1*	*TCF4*			*SLC13A5*
*KCNQ5*	*PRRT2*	*ZDHHC9*			*SLC2A1*
*KCNT1*	*SLC1A2*				*SLC35A2*
*KCTD7* ^a^	*SLC25A22*				*SLC35A3*
*LGI1*	*SYNGAP1*				*SLC6A8*
*SCN1A*					*SZT2*
*SCN1B*					*UBE3A*
*SCN2A*					*WWOX*
*SCN8A*					
*SLC12A5*					
*SLC6A1*					
*SLC9A6*					

CHE-45, CHE-76 additions, CHE-85 additions, CHE-102 additions

^a^Removed from CHE-102

### Sample preparation

Genomic DNA was extracted from blood using standard methods. For the CHE-45, panel libraries were prepared from 15 ng of template DNA using the Ion AmpliSeq library 2.0 kit and custom primers following the manufacturer’s instructions (ThermoFisher Scientific).^7^ The CHE-76, CHE-85 and CHE-102 panel libraries were prepared from 1000 ng template DNA, Agilent SureSelect target enrichment (Agilent technologies) and KAPA library preparation kit (KAPA Biosystems) following manufacturer’s instructions. The library DNA was clonally amplified onto the Ion Spheres Particles (ISPs) by emulsion PCR using an Ion OneTouch 2 system and the Ion PGM Template OT2 200 kit (ThermoFisher Scientific). ISPs were sequenced on an Ion PGM sequencer using an Ion 314, Ion 316 or Ion 318 chip and the Ion PGM 200 Sequencing kit as per the manufacturer’s instructions (ThermoFisher Scientific).

### Bioinformatics

Sequences were mapped to hg19 in the Torrent suite software (ThermoFisher Scientific) and variant calling was achieved in the Strand NGS software (Avadis) with a minimum of 20-fold read depth. Common SNPs with an allele frequency ≥ 2% and SNPs observed in more than 2 samples for each analyzed sample batch were filtered out. Genetic nonsynonymous/splice site variants were evaluated through database searches: dbSNP, Exome Variant Server, the Exome Aggregation Consortium database (ExAC), the Genome Aggregation Database (gnomAD) and HGMD Professional. Missense variants were also submitted to prediction softwares such as SIFT and PolyPhen-2, while splice site variants were evaluated by NNSPlice and Splicesite finder. Variants analyzed under a dominant inheritance model that were observed more than 10 times in ExAC were considered too common as monogenic causes. Potentially pathogenic variants were validated through conventional Sanger sequencing, and, if possible, parents were included for segregation analysis when indicated.

### Criteria for assessing pathogenicity of rare variants

We share the brief clinical summary of the patient with the laboratory to aid genotype-phenotype correlation; subsequently we interpret the gene panel report in detailed clinical context at a monthly multidisciplinary meeting including epileptologists (EH, REW, KL, DKP), a clinical neurophysiologist (SG) and genetic counselor (SO). We also consulted bioinformatics databases, patient registries, expert colleagues and published literature. Laboratory reported variants categorized by the ACMG system^11^ were then (re-)classified by us as either benign variants, VUS, or pathogenic variants for the purposes of genetic counselling. For predicted possibly damaging variants where segregation analysis could be performed, we required the variant to meet one of the following criteria to constitute a likely pathogenic variant: de novo in early-onset severe epilepsy syndromes, segregation with the disorder, inheritance from an unaffected parent but previously reported in other families with the same phenotype and incomplete penetrance, or adherence to a recessive X-linked or parent-of-origin mode of inheritance.

### Result feedback

We offered either a telephone or face-to-face consultation to the family, followed up with a written summary of the discussions in a letter.

### Opinion survey

We solicited the views of all 40 families through an anonymous 16-item questionnaire available as paper copy or web version (www.surveymonkey.com). The questionnaire covered three main topics of quality, impact and perceived value, and was formulated with the assistance of the Head of Patient Experience at one of the tertiary centres. We also sent an email link to a 10-item anonymous (www.surveymonkey.com) questionnaire to all 15 clinicians who had referred patients to the epilepsy genetics service (questions were adapted from a longer survey used in the evaluation of *SCN1A* testing^6^).

### Investigational cost

We searched electronic patient records to generate a list and timing of all investigations ordered in the neonatal epilepsy group; then matched these against 2017 hospital tariffs, separating them into categories of neuroimaging; EEG; routine blood tests; metabolic investigations of blood, urine and CSF; tissue biopsy; array CGH and karyotype; single gene tests; and NGS panel. We assessed the independent association of imaging, EEG, metabolic and genetic tests with diagnostic delay in days using multiple linear regression.

### Data availability

All supporting data can be found as presented in this paper.
